# Systems Biology Genetic Approach Identifies Serotonin Pathway as a Possible Target for Obstructive Sleep Apnea: Results from a Literature Search Review

**DOI:** 10.1155/2017/6768323

**Published:** 2017-09-19

**Authors:** Ram Jagannathan, Azizi Seixas, David St-Jules, Lakshmanan Jagannathan, April Rogers, Lu Hu, Girardin Jean-Louis, Mary Ann Sevick

**Affiliations:** ^1^Hubert Department of Global Health, Emory Global Diabetes Research Center, Atlanta, GA, USA; ^2^NYU School of Medicine, Department of Population Health, Center for Healthful Behavior Change, New York, NY 10016, USA; ^3^Vantage Research Center, Chennai 600 006, India

## Abstract

**Rationale:**

Overall validity of existing genetic biomarkers in the diagnosis of obstructive sleep apnea (OSA) remains unclear. The objective of this systematic genetic study is to identify “novel” biomarkers for OSA using systems biology approach.

**Methods:**

Candidate genes for OSA were extracted from PubMed, MEDLINE, and Embase search engines and DisGeNET database. The gene ontology (GO) analyses and candidate genes prioritization were performed using Enrichr tool. Genes pertaining to the top 10 pathways were extracted and used for Ingenuity Pathway Analysis.

**Results:**

In total, we have identified 153 genes. The top 10 pathways associated with OSA include (i) serotonin receptor interaction, (ii) pathways in cancer, (iii) AGE-RAGE signaling in diabetes, (iv) infectious diseases, (v) serotonergic synapse, (vi) inflammatory bowel disease, (vii) HIF-1 signaling pathway, (viii) PI3-AKT signaling pathway, (ix) regulation lipolysis in adipocytes, and (x) rheumatoid arthritis. After removing the overlapping genes, we have identified 23 candidate genes, out of which >30% of the genes were related to the genes involved in the serotonin pathway. Among these 4 serotonin receptors SLC6A4, HTR2C, HTR2A, and HTR1B were strongly associated with OSA.

**Conclusions:**

This preliminary report identifies several potential candidate genes associated with OSA and also describes the possible regulatory mechanisms.

## 1. Introduction

According to the Centers for Disease Control and Prevention, sleep disorders now constitute a public health epidemic. The National Healthy Sleep Awareness Project estimated that at least 25 million American adults are affected by OSA for the year 2014 [1]. The underlying pathophysiology and physiological mechanisms of OSA are multifactorial, may vary considerably between individuals, and are not clearly understood [2]. It is estimated that majority of the patients with sleep apnea are undiagnosed [3] increasing evidence supporting an association between OSA and obesity [4], hypertension [5], metabolic syndrome [6], type 2 diabetes (T2DM) [7] and cardiovascular mortality [8], increased risk of psychiatric disorders [9], and traffic and occupational accidents [10]. Given the potentially serious consequences of OSA, timely diagnosis and recognition, risk stratification, and appropriate treatment are of utmost importance [11].

In-lab polysomnography (PSG) is the “gold standard” test for the diagnosis of OSA [12]. However, PSG is resource-intensive and cumbersome and requires patients to spend the night under observation in a foreign (hospital) environment. Although home-based sleep monitoring devices overcome these problems, they are less accurate, are prone to technical breakdown, fail to capture other forms of sleep disorders, and underestimate the severity of OSA [13]. Recently, several sleep based psychometric questionnaires are available to screen individuals at low to high OSA risk. However, the lack of high quality evidence from large scale epidemiological studies hinders their applicability in clinical practice [12]. Also, questionnaires did not allow reliable differentiation between different phenotypes of OSA; that is, they were unable to identify individuals who are susceptible to metabolic, cardiovascular, and neurologic effects of OSA. Also, sleep based questionnaires may not be suitable to the general population because they include questions related to sleepiness and not all patients, even those with severe OSA, report sleepiness. For instance, Wisconsin Sleep Cohort Study found that only 37% of patients with severe OSA (Apnea-Hypopnea Index (AHI) score ≥ 30 events/h) are aware of questions related to daytime sleepiness [14].

Blood biomarkers may offer alternative approach for screening and identifying individuals at risk of developing OSA and related outcomes. Biomarker is defined as a characteristic that is objectively measured and evaluated as an indicator of normal biologic processes, pathogenic processes, or pharmacologic response to a therapeutic intervention. In the case of OSA, the candidate biomarkers should be useful for the following: diagnosis, assessing disease burden and severity, and evaluating response to treatment. Several epidemiological studies suggest that the circulatory levels of natriuretic peptide (BNP) or N-terminal-pro-BNP [15], leptin, C-reactive protein (CRP) [16, 17], TNF*α* [18], vaspin [19], ghrelin [20], and interleukin-6 [17] are associated with OSA. To ensure diagnostic accuracy, a biomarker should yield both high sensitivity and specificity to identify individuals at high risk of developing OSA. Such diagnostic performance would limit the necessity of PSG, an expensive and laborious modality, at least in some patients. Additional aspects should include low cost and facility in use, as well as the ability to simultaneously evaluate multiple pathogenic pathways. However, current knowledge of biomarkers for OSA is limited by data collection techniques, disease complexity, and potential confounding factors.

Advances in computational approaches and experimental omics methods that allow the simultaneous analysis of multidimensional data such as DNA, RNA, and proteins in a single analysis have made these systems biology approaches feasible for biomarker discovery [21]. Systems biology is a scientific discipline that endeavors to quantify all of the molecular elements of a biological system to assess their interactions and to integrate that information into graphical network models [21]. Therefore, the objective of this report is to (a) identify “novel” biochemical markers and associated pathways of OSA using systems biology approach, (b) assess the association of the selected candidate genes with OSA comorbid conditions, and (c) study potential interaction of the candidate genes in a biological network context.

## 2. Methodology

### 2.1. Literature Mining and Identification of Candidate Genes

Literature search was conducted in PubMed, MEDLINE, and Embase search engines for studies published for “genes, polymorphisms, and biomarkers” using the terms “Apnea, Obstructive Sleep” or “Apneas, Obstructive Sleep” or “Obstructive Sleep Apnea” or “Obstructive Sleep Apneas” or “Obstructive Sleep Apnea Syndrome” or “OSAHS” or “Sleep Apnea Hypopnea Syndrome” or “Sleep Apneas, Obstructive” or “Sleep Apnea Syndrome, Obstructive” or “Syndrome, Obstructive Sleep Apnea” or “Syndrome, Sleep Apnea, Obstructive” or “Syndrome, Upper Airway Resistance, Sleep Apnea” or “Upper Airway Resistance Sleep Apnea Syndrome” for obstructive sleep apnea (MeSH® ID: D020181). Furthermore, we queried the DisGeNET database (http://www.disgenet.org/web/DisGeNET/menu) [22] that integrates information from four repositories (search term: umls:C0520679 for OSA): (a) Online Mendelian Inheritance in Man (OMIM), (b) UniProt/SwissProt (UniProt), (c) Pharmacogenomics Knowledge Base (PHARMGKB), and (d) Comparative Toxicogenomics Database (CTD). DisGeNET can be accessed through the Cytoscape 2.8.3, a platform for complex network analysis. The identified genes were then evaluated to verify authenticity and to remove redundancies. Genes with unknown functions were excluded from the analysis (<5%).

### 2.2. Gene-Set Enrichment Analysis and Candidate Gene Prioritization

The gene ontology analysis was performed using ToppGene suite [23]. The ToppGene suite is used for (i) gene list functional enrichment, (ii) candidate gene prioritization using either functional annotations or network analysis, and (iii) identification and prioritization of novel disease candidate genes in the interactome. The disease pathways prioritization and gene enrichment analysis were performed using Enrichr (http://amp.pharm.mssm.edu/Enrichr/) [24, 25]. Enrichr is an integrative web-based and mobile software application that includes new gene-set libraries, an alternative approach to rank enriched terms, and various interactive visualization approaches to display enrichment results using the JavaScript library and Data Driven Documents (D3). Enrichr implements three approaches to compute enrichment: the first method is a standard Fisher exact test; the second method is Fisher exact correction test based on intuition analysis to generate a *z*-score of the deviation from the expected rank; and the third method (which we used for this analysis) is a combination of both *P* value computed using the Fisher exact test and the *z*-score. For this analysis, we have employed third approach for gene ontology analysis.

### 2.3. Network Analysis by Ingenuity Pathway Analysis (IPA)

Finally, the shortlisted genes were submitted to Ingenuity Pathway Analysis (IPA 4.0, Ingenuity Systems Inc., https://www.ingenuity.com/) for mapping to canonical pathways and identifying upstream regulators using a set of criteria: genes and endogenous chemicals, direct and indirect interactions, maximum molecules per network, networks per analysis, humans as the selected species, and all tissues and primary cells. The resulting networks were scored based on the fold change provided by Cuffdiff as log2 (fold change) for each gene. The obtained *P* values correspond to Fisher's exact test, with the null hypothesis that the molecules within the networks are connected based on chance.

## 3. Results: Candidate Gene Identification

**Figure 1:**
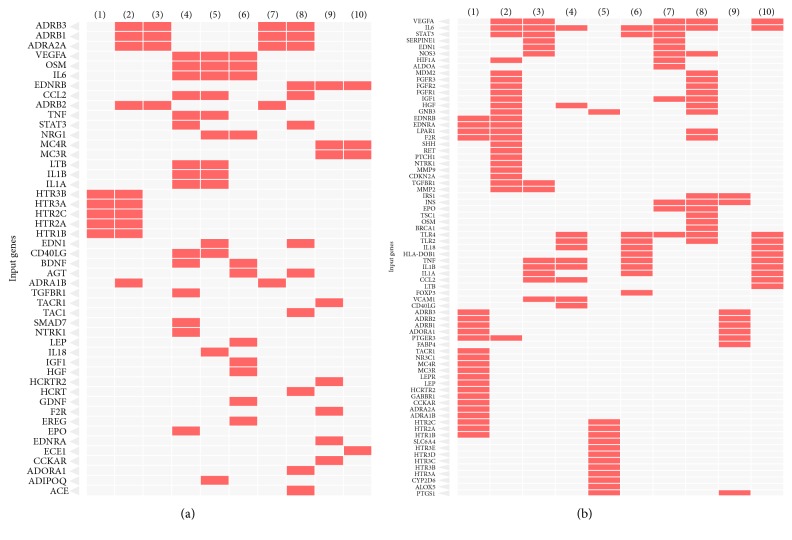
(a) Clustergram showing the top 10 highly significant gene ontology molecular functions associated with OSA. The selected genes (*n* = 153) were imported into Enrichr web tool using default setting. The top GO molecular activity was ranked based on enrichment scores (combined Fisher's exact test *P* values and the *z*-scores). The top 10 associated molecular functions are (1) serotonin receptor activity, (2) G-protein coupled amine receptor, (3) catecholamine binding, (4) cytokine receptor binding, (5) cytokine activity, (6) growth factor activity, (7) adrenergic receptor activity, (8) G-protein coupled receptor, (9) G-protein coupled peptide receptor, (10) and peptide hormone binding. (b) Clustergram showing the top 10 highly significant KEEG pathways associated with OSA. The selected genes (*n* = 153) were imported into Enrichr web tool using default setting. The top KEEG pathways were ranked based on enrichment scores (combined Fisher's exact test *P* values and the *z*-scores). The top 10 highly significant OSA disease pathways include (1) serotonin receptor interaction, (2) pathways in cancer, (3) AGE-RAGE signaling in diabetes, (4) infectious diseases, (5) serotonergic synapse, (6) inflammatory bowel disease, (7) HIF-1 signaling pathway, (8) PI3-AKT signaling pathway, (9) regulation lipolysis in adipocytes, and (10) rheumatoid arthritis.

**Figure 2:**
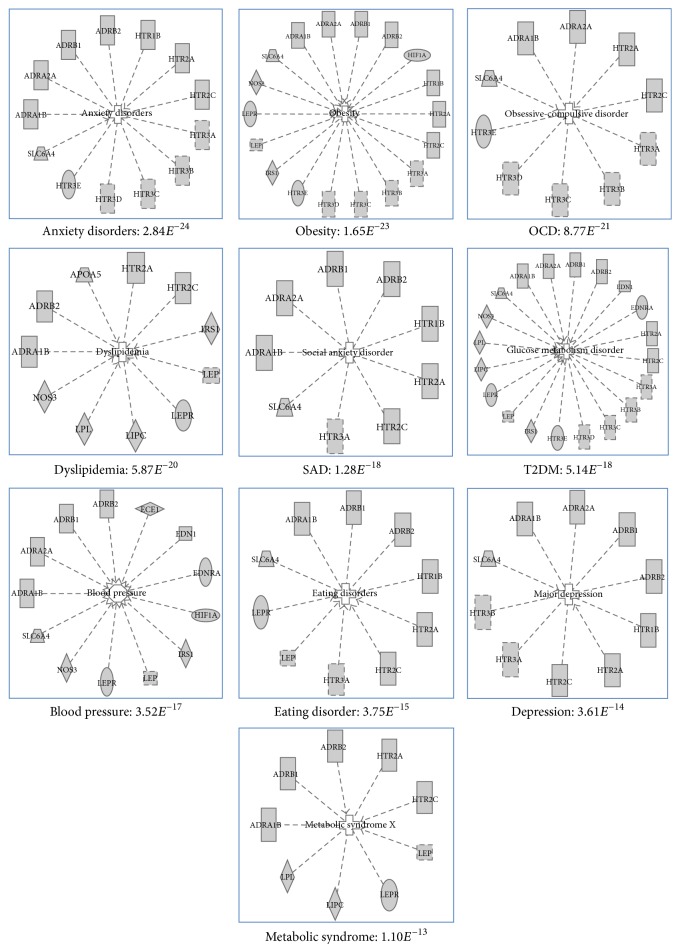
The genes identified from the enrichment analysis were significantly associated with OSA comorbidities. Ingenuity Pathway Analysis was used to create gene-disease network. The selected candidate genes (*n* = 23) were significantly associated with anxiety disorders (2.8*E*^−24^), obesity (1.65*E*^−23^), obsessive-compulsive disorder (8.77*E*^−21^), dyslipidemia (5.87*E*^−20^), social anxiety disorder (1.28*E*^−18^), type 2 diabetes (5.14*E*^−18^), blood pressure (3.25*E*^−17^), eating disorder (3.75*E*^−15^), depression (3.61*E*^−14^), and metabolic syndrome (1.10*E*^−13^).

**Figure 3:**
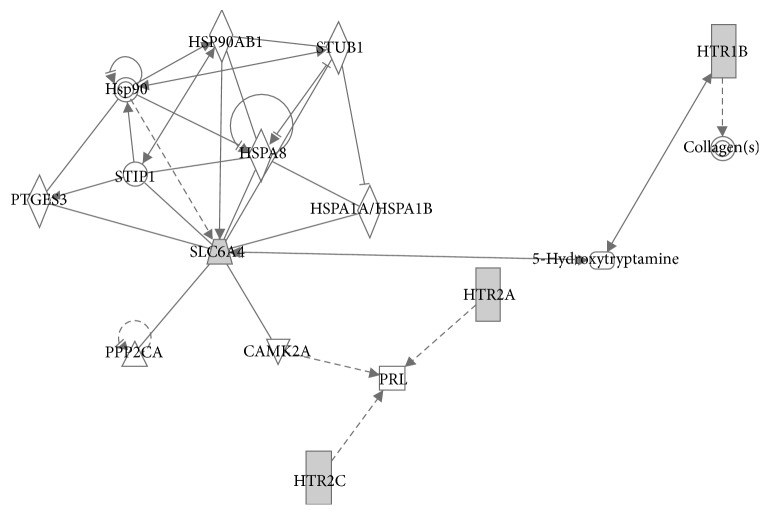
Network analysis found four serotonin receptors, HTR1B, SLC6A4, HTR2A, and HTR2C, significantly associated with OSA. Ingenuity Pathway Analysis was used to create a network analysis. Links of validated genes (in grey) and other genes or molecules are represented with a continuous (direct interaction) or discontinuous line (indirect interaction). The expression of our candidate genes could be modulated mainly by 3 molecules: 5-hydroxytryptamine (for HTR1B and SLC6A4), PRL (HTR2A and HTR2C), and HSP90AB1 (for SL6CA4).

Literature mining for the search terms as described in the methodology resulted in 2200 abstracts. After carefully screening the abstracts we got 550 genes related to OSA and from that we have shortlisted 153 genes (removal of redundancies, duplicates, unknown functions, etc.), for further analysis. The analysis of statistically overrepresented pathways in the shortlist of genes revealed 28 canonical pathway maps (by ToppGene suite) with confidence level *P* < 0.05 (adjusted for false discovery rate (FDR)). The top 10 pathways and their corresponding genes revealed by Enrichr tool are given in Figures 1(a) and 1(b). This includes (i) serotonin receptor interaction, (ii) pathways in cancer, (iii) AGE-RAGE signaling in diabetes, (iv) infectious diseases, (v) serotonergic synapse, (vi) inflammatory bowel disease, (vii) HIF-1 signaling pathway, (viii) PI3-AKT signaling pathway, (ix) regulation lipolysis in adipocytes, and (x) rheumatoid arthritis. After merging all the pathways, “23” unique genes remained for the core analysis (Box 1). The set of unique genes implicated in OSA showed maximal association with OSA comorbid conditions (Figure 2) such as anxiety disorders (*P* = 2.84*E*^−24^), obesity (*P* = 1.65*E*^−23^), obsessive-compulsive disorder (*P* = 8.77*E*^−21^), dyslipidemia (*P* = 5.87*E*^−20^), social anxiety disorder (*P* = 1.28*E*^−18^), T2DM (*P* = 5.14*E*^−18^), blood pressure (*P* = 3.52*E*^−17^), eating disorders (*P* = 3.75*E*^−15^), depression (*P* = 3.61*E*^−14^), and metabolic syndrome (*P* = 1.10*E*^−13^) (Figure 3). Among twenty-three genes identified, 9 (36.0%) genes are involved in serotonin receptor mediated pathways. Therefore, for the subsequent network analysis we focused only on serotonin pathways.

For network analysis Figure 3 shows the Ingenuity Pathway Analysis of the selected candidate genes and their corresponding molecular interactions. The significant network associations include (a) carbohydrate metabolism, small molecule biochemistry, and psychological disorders; (b) behavior, cell signaling, and neurological disease; and (c) drug metabolism, endocrine system development and function, and lipid metabolism. In the predicted merged network generated by IPA algorithm, four serotonin receptors, HTR1B, SLC6A4, HTR2A, and HTR2C, were found to be the potential candidates regulating the OSA. The expression of our candidate genes could be modulated mainly by 3 molecules: 5-hydroxy tryptamine (for HTR1B and SLC6A4), PRL (HTR2A and HTR2C), and HSP90AB1 (for SL6CA4). The important regulators of these genes were insulin (*P* < 1.05*E*^−09^), melatonin (*P* < 1.41*E*^−09^), glucocorticoid (*P* < 5.83*E*^−09^), lithium (*P* < 1.45*E*^−08^), and RAB1A (*P* < 5.48*E*^−08^).

## 4. Discussion

The overall validity of existing biomarkers in the diagnosis of obstructive sleep apnea (OSA) remains unclear. Although the existing adipokines and inflammatory markers seem to have a favorable potential to become a good biomarker to identify OSA they are not specific for the disease. In this study, we have performed the gene mining analyses and found 23 candidate genes that are likely associated with OSA. Functional enrichment analysis suggested that most of these genes are directly involved in the above-mentioned biological processes (Figure 2), which are highly relevant for OSA. Several pathways found in our study have already been discussed by many authors with inconclusive evidence [26–33] before as well as their involvement in the pathogenesis of OSA. For instance, Diefenbach et al. [34] showed the positive association between EDN1 variant Lys198Asn genotype and OSA. However, plasma levels of EDN1 were not associated with the OSA severity.

The disease pathway analysis showed that the selected candidate genes were also associated with the OSA comorbid conditions such as anxiety disorders, obesity, and dyslipidemia. For the subsequent analysis, we focused only on serotonin pathway because majority of serotonin genes make up this pathway. Dysfunction of the serotoninergic system has long been suspected in sleep disorders and respiratory diseases [35, 36]. 5-HT acts as both a neurotransmitter and a neuromodulator in the human central nervous system (CNS). Serotonin pathway affects food intake, sleep, anxiety, sexual behavior, and mood [37]. Approximately 2% of the body's serotonin is stored in central nervous system (CNS) and the rest of the body's serotonin is in the gut and stored peripherally, where it operates as a peripheral hormone, affecting vasoconstriction, intestinal motility, primary hemostasis, liver repair, and the control of the T-cell-mediated immune system [38]. Emerging evidence from animal studies showed that both peripheral and central 5-HT and its receptors were involved in sleep regulation [39]. The oxygen deprivation and hyperventilation which are characteristic of OSA can be mimicked and induced in panic attack which might derange the serotonin pathway mediators. Recently, 5-HT system has been shown to be involved in circadian regulation [40], glucose and lipid metabolism, and adipocyte differentiation [41, 42]. Serotonergic neurons have a significant influence on sleep/wake cycles due to their multiple connections throughout the cortex, basal forebrain, limbic system, and brainstem areas. The 5-HT systems function predominantly to promote wakefulness and inhibit rapid eye movement (REM) sleep. The recent finding suggests that serotonergic connections have effects on numerous CNS processes, and dysfunction in this system may be implicated in respiratory pathology such as OSA [43]. Serotonin is thought to cause its effects through these membrane-bound receptors [44]. In our analyses, we have identified 4 receptors: SLC6A4, HTR2_C_, HTR2_A_, and HTR1_B_ which associated with OSA.

The genomewide association studies demonstrated the association of 5-HTR 2A/2C polymorphisms with OSA. Furthermore, by regulating the magnitude and duration of serotonergic responses, the SLC6A4 (i.e., 5-HTT) is pivotal to the fine-tuning of brain serotonergic neurotransmission and of the peripheral actions of 5-HT [45].

## 5. Conclusion

It is clear that there is an intricate network of interacting genes regulating sleep and the derangement in them leads to OSA. In this preliminary report, we identified 23 novel biomarkers especially from the serotonin pathway which might associate with OSA. Consequently, we demonstrated network-level analysis of potential OSA candidate genes and the plausible regulatory mechanisms. Limited evidence from the clinical trials showed that 5-HT has a critical role influencing respiratory control during sleep. 5-HT acts as a potent central ventilatory stimulant and serves to maintain upper airway patency and maintain eucapnia via the chemoreceptor properties. Relative reductions in 5-HT may lead to the development and worsening of OSA. Further biological experiments are warranted to study the role of peripheral 5-HT and its receptors on the pathogenesis of OSA.

## Figures and Tables

**Box 1:**
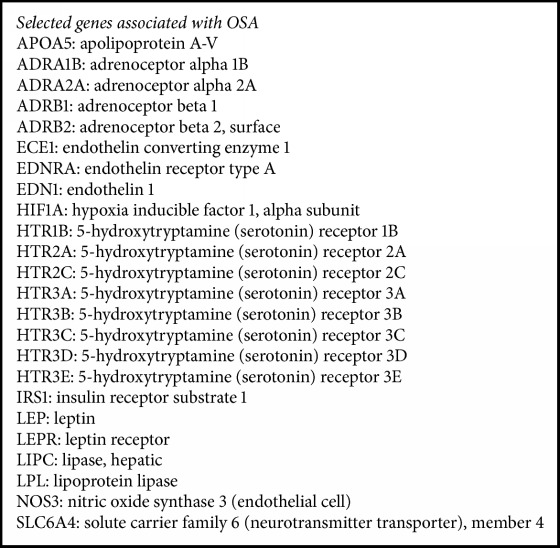
Top 23 candidate genes identified by enrichment analysis. The box shows the 23 unique genes (in alphabetical order) identified from Enrichr analysis. The majority of the pathway genes are involved in serotonin based regulatory pathways.
